# Supplementary figures and images for: Association between the shock index on admission and in-hospital mortality in the cardiac intensive care unit
Source: PLoS One. 2024 Apr 16;19(4):e0298327. doi: 10.1371/journal.pone.0298327 (PMC11020967; doi:10.1371/journal.pone.0298327)

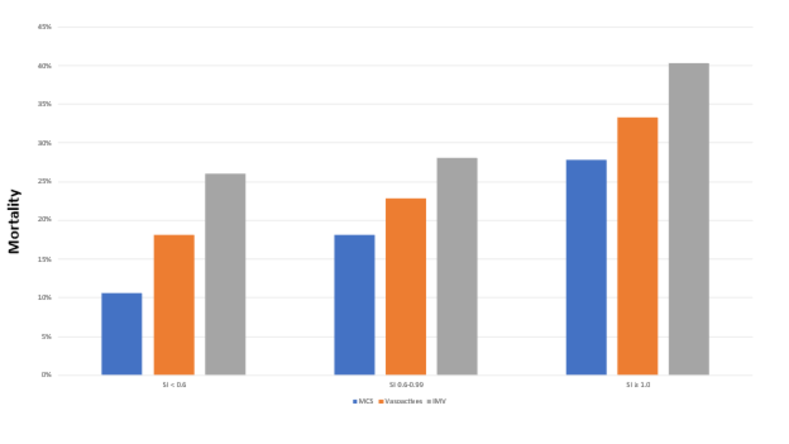

Supplement: S1 Fig — (TIF) [file pone.0298327.s001.tif]

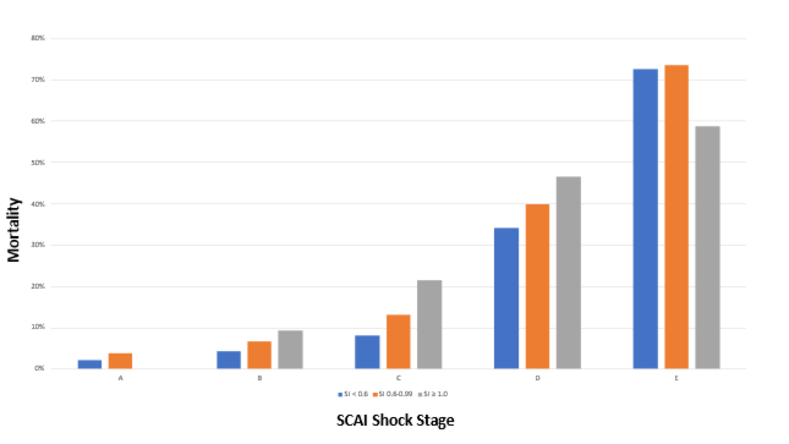

Supplement: S2 Fig — (TIF) [file pone.0298327.s002.tif]

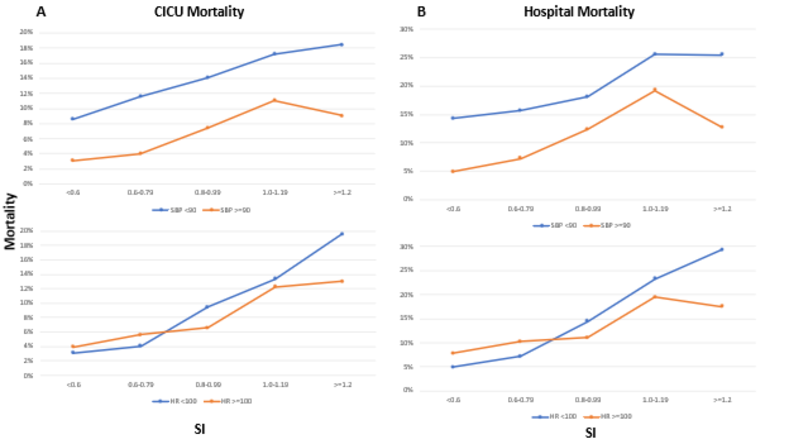

Supplement: S3 Fig — A: Line graph demonstrating the relationship of SI stratified by HR and SBP on CICU mortality. B: Line graph demonstrating the relationship of SI stratified by HR and SBP on hospital mortality. (TIF) [file pone.0298327.s003.tif]

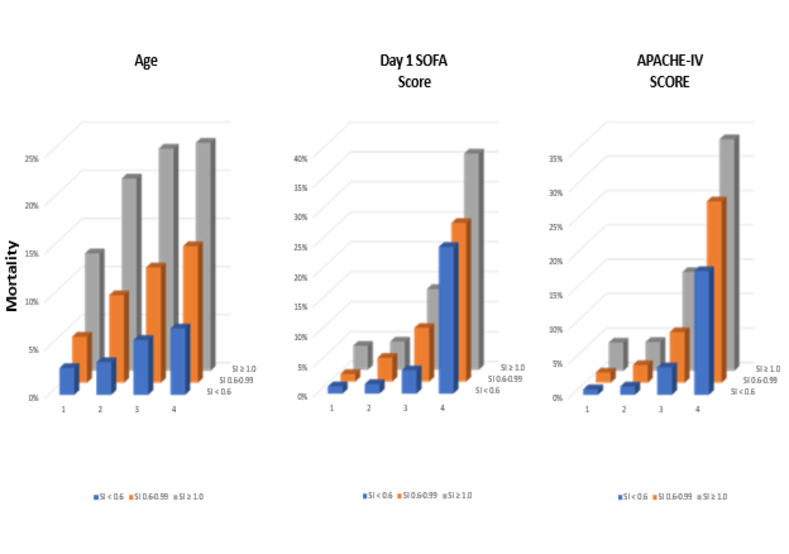

Supplement: S4 Fig — (TIF) [file pone.0298327.s004.tif]
