# Supplementary material for: Association between the shock index on admission and in-hospital mortality in the cardiac intensive care unit
Source: PLoS One. 2024 Apr 16;19(4):e0298327. doi: 10.1371/journal.pone.0298327 (PMC11020967; doi:10.1371/journal.pone.0298327)
Supplement: S1 Table — Data from Jentzer, et al. J Am Coll Cardiol 2019. (DOCX) [file pone.0298327.s005.docx]

**Supplemental Table 1:**  Study definitions of hypotension, tachycardia, hypoperfusion, deterioration and refractory shock. Data from Jentzer, et al. J Am Coll Cardiol 2019.

| **Term** | **Definition** |
| --- | --- |
| **Hypotension/tachycardia** | **Presence of any of the following criteria:**   - Admission systolic BP <90 mmHg - Minimum systolic BP <90 mmHg during first 1 hour - Admission MAP <60 mmHg - Minimum MAP <60 mmHg during first 1 hour - Admission HR >100 BPM - Maximum HR >100 BPM during first 1 hour - Admission HR > admission systolic BP - Mean HR > mean systolic BP during first 1 hour |
| **Hypoperfusion** | **Presence of any of the following criteria:**   - Admission lactate >2 mmol/L - Urine output <720 ml during first 24 hours - Creatinine increased by ≥0.3 mg/dl during first 24 hours |
| **Deterioration** | **Presence of any of the following criteria:**   - Maximum lactate > admission lactate - # vasoactives during first 24 hours > # vasoactives during first 1 hour - Maximum VIS during first 24 hours > VIS during first 1 hour - Maximum NEE during first 24 hours > NEE during first 1 hour |
| **Refractory shock** | **Presence of any of the following criteria:**   - Mean systolic BP during first 1 hour <80 and on vasoactives - Mean MAP during first 1 hour <50 and on vasoactives - # vasoactives during first 1 hour >2 - # vasoactives during first 1 hour >1 and IABP during first 24 hours - Admission lactate ≥10 mmol/L |

**Abbreviations:** BP, blood pressure; HR, heart rate; IABP, intra-aortic balloon pump; MAP, mean arterial pressure; NEE, norepinephrine-equivalent vasopressor dose; VIS, Vasoactive-Inotropic Score.

- **VIS** is calculated as using vasoactive drug doses (in mcg/kg/min), as follows: VIS = dobutamine + dopamine + (10 * phenylephrine + milrinone) + (100 * [epinephrine + norepinephrine]) + (10000 * units/kg/min vasopressin).
- **NEE** is calculated using the dose equivalency as follows: 0.1 mcg/kg/min norepinephrine = 0.1 mcg/kg/min epinephrine = 15 mcg/kg/min dopamine = 1 mcg/kg/min phenylephrine = 0.04 U/min vasopressin.
