# Supplementary material for: Association between the shock index on admission and in-hospital mortality in the cardiac intensive care unit
Source: PLoS One. 2024 Apr 16;19(4):e0298327. doi: 10.1371/journal.pone.0298327 (PMC11020967; doi:10.1371/journal.pone.0298327)
Supplement: S2 Table — Data from Jentzer, et al. J Am Coll Cardiol 2019. (DOCX) [file pone.0298327.s006.docx]

**Supplemental Table 2:** Definition of cardiogenic shock (CS) stages used in this study, based on the Society for Cardiovascular Angiography and Intervention (SCAI) consensus statement classification. Data from Jentzer, et al. J Am Coll Cardiol 2019.

| **Cardiogenic shock stage** | **SCAI Definition** |
| --- | --- |
| **Stage A (“At risk”)** | Patients **without** CS who are hemodynamically stable but have acute cardiovascular disease putting them at risk of developing CS |
| **Stage B (“Beginning”)** | Patients **without** CS who display hemodynamic instability, including hypotension and/or tachycardia, but with normal perfusion |
| **Stage C (“Classic”)** | Patients **with** CS, manifested by hypoperfusion (lactic acidosis, oliguria, cool/clammy periphery or altered mentation) requiring intervention |
| **Stage D (“Deteriorating)”** | Patients **with CS** whose hemodynamic instability and/or hypoperfusion fails to respond to initial interventions |
| **Stage E (“Extremis”)** | Patients **with CS** and overt or impending circulatory collapse, including CA with ongoing resuscitation |
