# Supplementary material for: Association between the shock index on admission and in-hospital mortality in the cardiac intensive care unit
Source: PLoS One. 2024 Apr 16;19(4):e0298327. doi: 10.1371/journal.pone.0298327 (PMC11020967; doi:10.1371/journal.pone.0298327)
Supplement: S3 Table — (DOCX) [file pone.0298327.s007.docx]

| **Supplemental Table 3** |
| --- |
| **Variables** |
| Age |
| Female |
| Invasive Ventilation |
| CCI |
| SOFA Score |
| VIS |
| Dialysis |
| PCI |
| Angiogram |
| Cardiac Arrest |
| Shock |
| Respiratory Failure |
| Braden Skin Score |
| In-hospital arrest |
| SCAI Shock Stage |
