# Supplementary material for: Association between the shock index on admission and in-hospital mortality in the cardiac intensive care unit
Source: PLoS One. 2024 Apr 16;19(4):e0298327. doi: 10.1371/journal.pone.0298327 (PMC11020967; doi:10.1371/journal.pone.0298327)
Supplement: Attachment — Submitted filename: Response to Reviewers.docx [file pone.0298327.s008.docx]

**Response to Reviewers Comments**

**RE: PONE-D-23-31759. Association between the shock index on admission and in-hospital mortality in the cardiac intensive care unit.**

**To:**

**Dr. Gaetano Santulli, M.D., Ph.D., FAHA**

**Academic Editor. PLOS ONE**

**Dear Dr. Santulli,**

**Thank you for the interest in this original research manuscript. We believe this manuscript will be of interest to the audience of PLOS ONE based on the simplicity of using the shock index as an early risk stratification and prognostication tool in the cardiac intensive care unit.**

**We appreciate the thoughtful comments of the Reviewers and have addressed them in bold point-by-point format below in our response letter. We have made many adjustments and improvements to the manuscript to conform to PLOS ONE’s style requirements and to enhance the clarity of the manuscript. The suggested changes have certainly strengthened the impact of our manuscript and we hope you will find it suitable for publication in its revised format.**

**Please do not hesitate to reach out with any additional questions or concerns.**

**Sincerely,**

**Mitch Padkins, M.D.**

**On behalf of the authors.**

**Journal Requirements**

1. Please ensure that your manuscript meets PLOS ONE's style requirements, including those for file naming. The PLOS ONE style templates can be found at
   <https://journals.plos.org/plosone/s/file?id=wjVg/PLOSOne_formatting_sample_main_body.pdf> and 
   <https://journals.plos.org/plosone/s/file?id=ba62/PLOSOne_formatting_sample_title_authors_affiliations.pdf>

**Authors’ Response:** **The manuscript was edited in multiple areas to ensure that the manuscript meets PLOS ONE’s style requirements.**

2. We note that you have included the phrase “data not shown” in your manuscript. Unfortunately, this does not meet our data sharing requirements. PLOS does not permit references to inaccessible data. We require that authors provide all relevant data within the paper, Supporting Information files, or in an acceptable, public repository. Please add a citation to support this phrase or upload the data that corresponds with these findings to a stable repository (such as Figshare or Dryad) and provide and URLs, DOIs, or accession numbers that may be used to access these data. Or, if the data are not a core part of the research being presented in your study, we ask that you remove the phrase that refers to these data.

**Authors’ Response: The data was not a core part of the manuscript. Thus, this section was removed and the statement “data not shown” was removed.**

**Manuscript Excerpt:**

**Removed section: “In our cohort, we found a slightly higher area under the curve for discrimination of in-hospital mortality with the TIMI Risk Index than the SI in our cohort (0.68 versus 0.66, data not shown)”.**

3. Please include captions for your Supporting Information files at the end of your manuscript, and update any in-text citations to match accordingly. Please see our Supporting Information guidelines for more information: <http://journals.plos.org/plosone/s/supporting-information>.

**Authors’ Response: Captions for supporting information was added to the end of the manuscript in accordance with the Supporting Information Guidelines.**

**Manuscript Excerpt:**

**“Supporting information**

**S1 Fig. Bar graph demonstrating the relationship between mechanical support devices, vasoactive medications, and mechanical ventilation on mortality stratified by SI.**

**S2 Fig. Bar graph demonstrating the relationship between SCAI shock stage and mortality.**

**S3 Fig. A: Line graph demonstrating the relationship of SI stratified by HR and SBP on CICU mortality. B: Line graph demonstrating the relationship of SI stratified by HR and SBP on hospital mortality.**

**S4 Fig. Bar graph demonstrating the relationship of SI stratified by quartiles of age, day 1 SOFA score, and APACHE-IV score on mortality.**

**S1 Table. Study definitions of hypotension, tachycardia, hypoperfusion, deterioration and refractory shock. Data from Jentzer, et al. J Am Coll Cardiol 2019.**

**S2 Table. Definition of cardiogenic shock (CS) stages used in this study, based on the Society for Cardiovascular Angiography and Intervention (SCAI) consensus statement classification. Data from Jentzer, et al. J Am Coll Cardiol 2019.**

**S3 Table. Variables used for multi-variable logistic regression model”.**

**Reviewer Comments to the Author**

**Reviewer #1**

1. Reviewer #1: This study provides compelling evidence regarding the predictive ability of the shock index for mortality in predicting patients in the CICU. However, there are a few suggestions to consider. Firstly, regarding Figure 3A, it appears that the correlation between vital signs and mortality is not sufficiently clear, making it less intuitive for readers to understand the intended message. It is recommended to make moderate adjustments for clarity.

**Authors’ Response: We appreciate the Reviewer’s comment here. We had used the 3D bar graph format to make it easy for the reader to see that either a lower systolic BP or a higher HR incrementally predicts a higher mortality in an additive fashion. We considered a flat bar graph, but in our opinion this makes it a bit harder for the reader to perceive the additive nature of these two variables. Please see below for the Reviewer’s reference, we could replace Figure 3A with this if it is preferred.**

|  | >=110 | 90-109 | <90 |
| --- | --- | --- | --- |
| <90 | 3.08% | 5.24% | 13.33% |
| 90-109 | 5.46% | 8.88% | 21.03% |
| >=110 | 6.76% | 10.03% | 16.28% |

2. Secondly, Table 3 and Figure 5 seem to convey similar information, differing only in the presentation of shock index as a continuous variable or grouped discussion. I believe that grouping shock index as < 0.6, 0.6-0.99, and ≥ 1.0 may not have significant meaning in this context. Perhaps, it would be more meaningful to analyze shock index as a continuous variable directly. If the author agrees with this idea, I suggest using mortality and non-mortality as grouping criteria for Table 1 and Table 2

**Authors’ Response: We agree that these are complementary approaches to showing related data among important subgroups. It is our opinion that displaying the mortality information as both a continuous variable (Table 3) and categorized (Figure 5) provides the reader with an additive understanding of the relationship between SI and mortality in case there could be non-linearity. We selected these cut-offs from the literature, although admittedly there is not clear consensus regarding the ideal cut-offs. More importantly, using these categorized cut-offs has the important advantage of allowing us to describe the study population and how patients with lower versus higher shock index differ. In our opinion, showing how patients with low versus high SI differ (as we have currently provided in Tables 1 & 2) is important for screening for potential confounders between SI and outcomes. This would be lost if we are primarily comparing survivors and non-survivors in the tables. Accordingly, we hope the Reviewer will find it acceptable to keep our current figures and tables as is.**

**Reviewer #2**

1. Abstract: As this is non-speciality journal, spell out CICU and BPM.

**Authors’ Response: These acronyms are now spelled out in the abstract and at the first mention of these words in the main text.**

**Manuscript Excerpt:**

**Abstract**

**“We aimed to determine whether the SI can aid in mortality risk stratification in unselected cardiac intensive care unit patients”.**

**“After multivariable adjustment, both heart rate (adjusted OR 1.06 per 10 beats per minute higher; CI 1.02-1.10; p-value 0.005) and systolic blood pressure (adjusted OR 0.94 per 10 mmHg higher; CI 0.90-0.97; p-value < 0.001) remained associated with higher in-hospital mortality”.**

**Manuscript**

**“The contemporary cardiac intensive care unit (CICU) cares for a complex mix of patients with acute cardiovascular conditions and critical illness”.**

**“Among 10,004 unique CICU patients in our database, we included 9,939 with available data for SI. Median HR, SBP, and shock index for our cohort was 79 (67, 94) beats per minute (BPM), 121 (105, 139) mmHg, and 0.65 (0.63, 0.68) BPM/mmHg, respectively”.**

2. Methods (ln. #122-123): Why did the authors use “admission vital signs, clinical measurements, and laboratory values” at or after CICU admission? Prediction being the primary objective, it seems more common to use values before the event occurs.

**Authors’ Response: Recognizing that CICU providers may not have access to data from before CICU admission (e.g., outside hospital admissions), it is our opinion that examining the patient’s condition (i.e., vital signs) at the time of CICU admission is most pertinent and predicting future events (mortality) based on these data is the basis for most CICU outcomes research (including our own cited works). Even for early deaths, these events occurred after these first recorded vital signs.**

**Manuscript excerpt:**

**Methods**

**“The admission vital signs, clinical measurements, and laboratory values were defined as the first value recorded after or closest to the index CICU admission.”**

3. Methods (ln. #143-144): Was the outcome fully ascertained in this notification system?

**Authors’ Response: Yes, in-hospital mortality was fully ascertained using this validated notification system.**

**Manuscript excerpt:**

**Methods**

**“All-cause CICU and in-hospital mortality were determined using an electronic review of medical records for notification of patient death”.**

4. Results: Were there any patients with a device implemented for arrhythmia?

**Authors’ Response: Unfortunately, our data set does not have access to this information and we added this as a limitation in the manuscript.**

**Manuscript Excerpt:**

**“We did not have data regarding the presence of implantable electronic cardiac rhythm devices or anti-arrhythmic drugs, which could have influenced the HR and thus the SI and its association with mortality.”**

5. Results ((ln. #166)): Vasoactive agents were used in 7% of patient, but this figure was lower than those presented in Table 1 and 2.

**Authors’ Response: We appreciate the Reviewer’s question, but we want to clarify that 7% refers to pulmonary artery catheter use. Vasoactive agents were used in 25% of our cohort, as demonstrated in tables 1 and 2.**

**Manuscript Excerpt:**

**“Mechanical support devices, pulmonary artery catheter, and vasoactive medications were used in 931 (9%), 721 (7%), and 2,452 (25%) respectively”.**

6. Results (ln. #180-182): OR of 1.14 and 0.84 do not appear “strong” (at least to me).

**Authors’ Response: We respect the Reviewer’s opinion here. However, we want to note that these are unit odds ratios per 10-unit higher HR and SBP values, and patients with larger differences would have proportionally higher OR values.**

7. Results (ln. #202): ? BPM/mmHg.

**Authors’ Response: This was updated to reflect the units of the ratio.**

**Manuscript Excerpt:**

**“As SI increased, there was a progressive and incremental increase in in-hospital mortality (unadjusted OR 1.19 per 0.1 BPM/mmHg higher, CI 1.16-1.21, p < 0.001; Fig 4)”.**

8. Results (ln. #205): Spell out “MCS.”

**Authors’ Response: The acronym MCS was spelled out.**

**Manuscript Excerpt:**

**“A higher SI (particularly SI ≥1) was associated with increased in-hospital mortality across different admission diagnoses (Table 2, Fig 5) and in patients supported with invasive ventilation, vasoactive drugs, or mechanical circulatory support (S1 Figure)”.**

9. Discussion (ln. #307-310): Redundant as this was mentioned in Methods section.

**Authors’ Response: This section was taken out as it was already discussed in the Methods section.**

**Manuscript Excerpt:**

**Removed section: “Informed consent for this study was waived by the Mayo Clinic IRB due to the minimal risk nature of the study under the Declaration of Helsinki. According to Minnesota statue law statute 144.295, patients who did not provide written or verbal Minnesota Research Authorization were excluded from the study”.**

10. Conclusion (ln. #317): “stronger” as compared what?

**Authors’ Response: SI was found to be a better discriminator of mortality than using heart rate and systolic blood pressure alone or combined. This point was made clearer in the revised manuscript.**

**Manuscript Excerpt:**

**Conclusion:**

**“Overall, SI was found to be a better discriminator of mortality than using HR and SBP individually or combined”.**

**Reviewer #3**

1. More description about the multivariate model would be useful.

**Authors’ Response: We performed multivariable analysis using logistic regression as described in the Methods. We specifically selected covariates that were clinically relevant or previously demonstrated to have significant association with in-hospital mortality in our prior analyses from this CICU cohort. Because SI was one of the criteria used to define the SCAI Shock Stage, we ran two sets of multivariable models—with and without inclusion of this variable.**

**Manuscript excerpt:**

**Methods**

**“Odds ratio (OR) and 95% confidence interval (CI) values for in-hospital mortality were estimated using logistic regression before and after multivariable adjustment. Multivariable models were adjusted for the following variables selected *a priori* based on clinical relevance and known associations with in-hospital mortality in CICU patients: age, gender, invasive ventilation, CCI, SOFA score, peak VIS, dialysis, percutaneous coronary intervention (PCI), angiogram, cardiac arrest, shock, respiratory failure, Braden skin score, and in-hospital arrest; models were run both with and without SCAI shock stage as an additional variable”**

2. The adjusted data are mentioned in the results section, but seem like they would be helpful to include in the main figures of the paper as well.

**Authors’ Response: We appreciate the Reviewer’s perspective here, but in our opinion the crude/unadjusted mortality rate is more easily understood by readers and interpreted from the figures. Accordingly, we would prefer to retain the figures showing crude/unadjusted mortality. The primary motivation for providing the adjusted analyses was to demonstrate that observed differences in crude mortality according to vital signs were not driven solely by confounding, considering the differences between patients with and without abnormal vital signs. We hope that the Reviewer will agree with our perspective.**
